# Surgical anatomy of sigmoid sinus with evaluation of its venous dominance for advances in preoperative planning

**DOI:** 10.3389/fnins.2023.1161179

**Published:** 2023-04-27

**Authors:** Chunran Zhu, Yulong Chong, Chenjun Jiang, Wu Xu, Jing Wang, Chengrong Jiang, Weibang Liang, Bei Wang

**Affiliations:** ^1^Department of Neurosurgery, Nanjing Drum Tower Hospital Clinical College of Nanjing University of Chinese Medicine, Nanjing, China; ^2^Affliated Hospital of Integrated Traditional Chinese and Western Medicine, Nanjing University of Chinese Medicine, Nanjing, China; ^3^Department of Physics, Faculty of Science, The University of Auckland, Auckland, New Zealand; ^4^Department of Nursing, Affiliated Hospital of Integrated Traditional Chinese and Western Medicine, Nanjing University of Chinese Medicine, Nanjing, China

**Keywords:** MVD, sigmoid sinus, MRI, preoperative planning, side dominance

## Abstract

Microvascular decompression (MVD) is a widely adopted neurosurgery in treating cranial nerve diseases providing long-term pain relief. Improving surgical techniques has been a focus of recent studies. Venous structures such as the sigmoid sinus are essential to protect, and whose risk of destruction during surgery increases with size. The medical records of patients who went through MRI ahead of MVD surgery between Dec 2020 and Dec 2021 were reviewed. Section area of sigmoid sinus calculated from the MRI plane of auditory nerve showed a right dominance of the sinus. The improved method concerning the relationship between affected side and the dominant sigmoid sinus offered a better bone window and surgical field by planning the operation incision in advance. Intraoperative adjustment of the bone flap was avoided, and the risk of destructing the sigmoid sinus was reduced.

## Introduction

**Figure 1:**
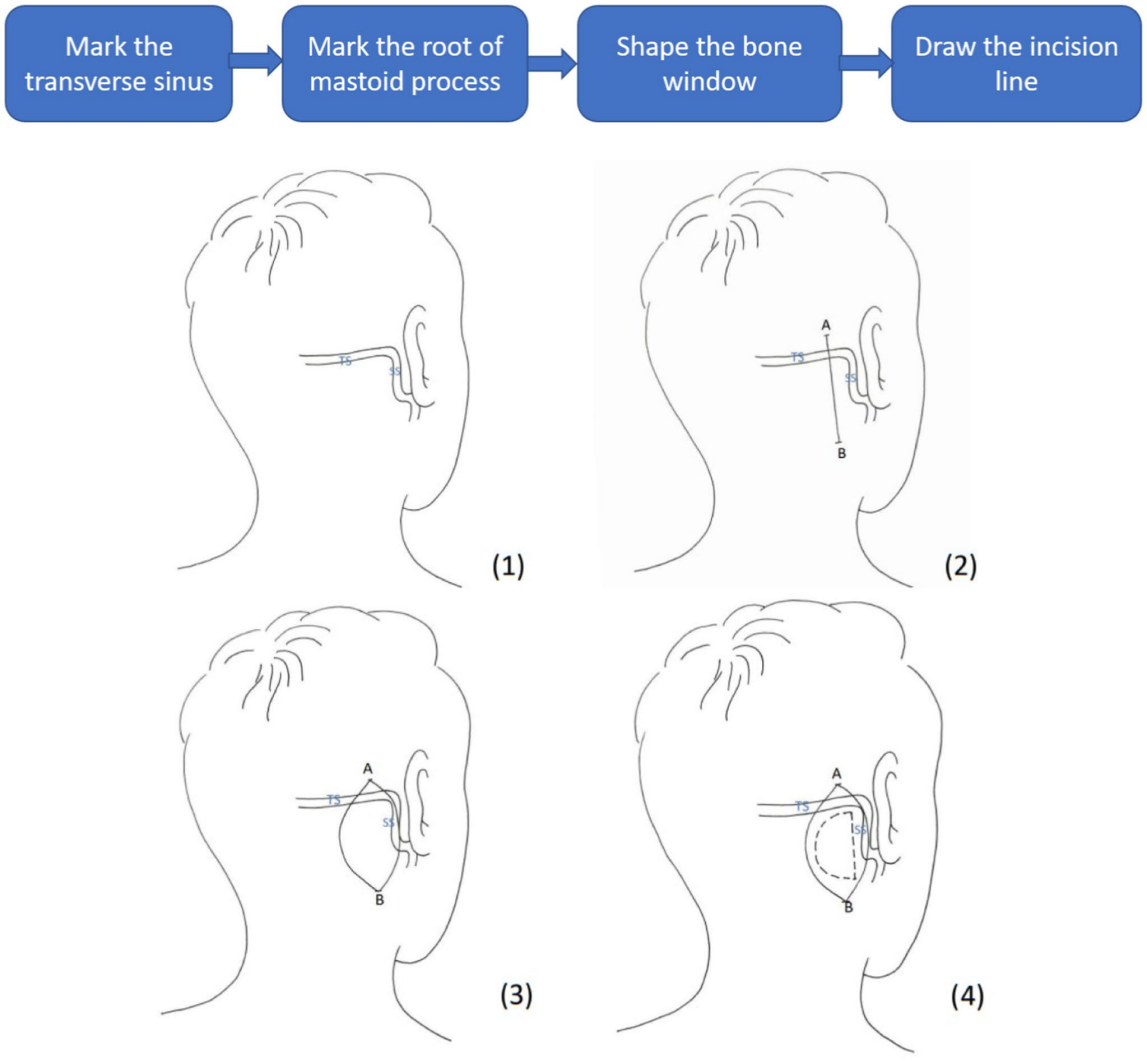
Conventional restrosigmoid access to reach the offending artery. TS, Transverse Sinus; SS, Sigmoid Sinus; line AB, line of incision; dashed line, D-shaped bone flap.

Microvascular decompression (MVD) is recognized as the gold standard treatment for cranial nerve diseases such as primary trigeminal neuralgia (PTN), primary hemifacial spasm (pHFS) and glossopharyngeal neuralgia (GN) (Montava et al., 2016; Patel and Liu, 2016; Apra et al., 2017; Min et al., 2019). After its proposal by Professor Gardner in the US, MVD spread to Japan and European countries and was considered one of the breakthrough technologies in modern treatment of neurological disorders (Møller, 1998). In the 1980s, experts in neurosurgery, represented by Professor Huanzong Zuo, pioneered the application of MVD in treating nerve diseases in China and promoted its use throughout the country. As the micro neurosurgical techniques develop, we know more about the pathogenesis of cranial nerve diseases. MVD surgery has been adopted to broader surgical areas including vestibulocochlear neurovascular compression syndrome, primary neurogenic hypertension, nervus intermedius neuralgia, spasmodic torticollis, and masseter spasm (Lee et al., 2019). Neurosurgeons have been pursuing a high cure rate and low occurrence of postoperative complications of MVD while technology advances. MVD operation involves separating the trigeminal ganglion from the offending artery that initially placed pressure on the trigeminal root leading to pain signals, and retrosigmoid craniotomy is frequently conducted in MVD surgery for approach the trigeminal ganglion (Van Osch et al., 2019). In the retrosigmoid approach, line of incision is drawn at the mastoid, and a bone flap is removed exposing the sigmoid sinus. The cerebellum is often pulled gently to achieve a better surgical view of the trigeminal neuron. This approach thus requires anatomical knowledge of the critical structures to ensure the success of surgery (Tomasi et al., 2020). A conventional procedure of a retrosigmoid approach for MVD surgery treating trigeminal neuralgia is shown in Figure 1. First, a line of connection is drawn between the mastoid process located posterior to the auricle and the external occipital protuberance to locate the transverse sinus as the upper edge of the bone flap. The position of the root of the mastoid process is then marked to locate the anterior edge of the bone flap. The bone flap is designed in a D shape, with an approximate length of 3 cm and width of 2.5 cm. The line of incision is drawn at the midline of the bone flap, with a length of about 5-7 cm. The anterior edge of the muscle separation is designed to reach the anterior abdominal groove, and the posterior edge to reach 0.5 cm behind the posterior edge of the bone flap. The procedure for the facial nerve MVD is similar to that of the trigeminal nerve MVD, but the incision should be lowered by 0.5-1 cm.

Venous structures closely monitored are the sigmoid sinus and transverse sinus, whose exposure during MVD craniotomy marks the completion of the bone flap. Meanwhile, protection of venous structures during surgery is vital. Any destruction of the venous structures, especially on the dominant side, may lead to devastating effects. Dominance pattern of the sigmoid sinus affects venous flow rate, flow volume and other characteristics of the sigmoid sinus which are essential to evaluate before conducting MVD. Surgery on the cranial side of a dominant venous sinus with a large sectional area increases the difficulty and risk of operation. Thus, it is necessary to evaluate the dominant sigmoid sinus and to plan for the dimensions of the bone flap before surgical interventions. X-ray, computed tomography (CT), and magnetic resonance imaging (MRI) and angiography (MRA) has been widely adopted in preoperative planning of MVD to provide morphometric information on the posterior fossa and the neurovascular structures of the trigeminal nerve (Toda et al., 2015). Meanwhile, pulling force on the cerebellum should be minimized to reduce tension on nerve bundles of the cerebellar hemisphere, which requires the bone flap design to provide a good surgical view. This study aims at advancing surgical techniques, especially preoperative planning methods, providing analysis of the dominance pattern of sigmoid sinus and MRI results.

## Materials and methods

### Cases information

We retrospectively analyzed the medical records of 278 patients who underwent MVD surgery at our institute between Dec 2020 and Dec 2021. Cross-sectional areas of the sigmoid sinus were calculated from the magnetic resonance plane of auditory nerve.

### Selection criteria of the cases

(1) Hospitalized due to primary trigeminal neuralgia or hemifacial spasm, (2) Received MVD surgical treatment, and (3). Underwent MRI examination before the surgery.

Cases of a second surgery and anatomical pathologies were excluded.

### Statistical analysis

Statistical analyses were carried out using SPSS 20.0 software. Results were presented in the form of average ± standard error (X ± S). Repeated measurement ANOVA was used for different times in the same measurement group. One-way analysis of variance was used to compare different groups. The t-test was used for inter-group or intra-group comparisons. A magnitude of t-value greater than or equal to 1.96 (i.e., |*t*| ≥ 1.96) indicates statistical significance. Data counts were tested by chi-square and index correlation was represented by Pearson correlation coefficient (Pearson’s r). A *p*-value less than 0.05 (i.e., *p* < 0.05) indicates statistical significance.

### Advance in surgical approach

The general line-drawing method was improved in later cases by designing the bone flap ahead of surgical interventions. The conventional method was to determine the position of the transverse sinus, then draw the line of surgical incision according to the location of the mastoid root. In the advanced approach, the relationship between the affected side and the side of the dominant sigmoid sinus was determined by MRI before surgery. Characteristics including the position, structure, and angle of the sigmoid sinus on the affected side and the thickness of the skull were evaluated by individuals to determine the position of surgical incision.

## Results

### Cases overview

278 patients aged 53.96 ± 10.88 years received an average hospitalization of 14.41 ± 4.25 days, characteristics shown in Table 1. Among them, 222 belonged to the ethnic Han, making up 79.9%; 56 belonged to other ethnicities, making up 20.1%. 87 of the patients (31.3%) were males and 191 (68.7%) were females. 145 patients presented with affections on the left and 133 on the right sides, making up 52.2 and 47.8%, respectively.

**Table 1:** Characteristics of 278 facial muscle spasm and trigeminal neuralgia cases.

Characteristic		*N* (%) or Mean ± SD
Age (year)		53.96 ± 10.88
Gender	Male	87 (31.3)
	Female	191 (68.7)
Nation	Han	222 (79.9)
	Other	0 (0.0)
	Missing	56 (20.1)
Diagnosis	Left	145 (52.2)
	Right	133 (47.8)
Length of hospitalization (day)		14.41 ± 4.25

### Results of analysis

Section areas of sigmoid sinus of 278 patients were measured from the magnetic resonance plane of auditory nerve. The left and right sigmoid sinus section area corresponding to different percentages of the sample are shown in Table 2. The section area of the right sigmoid sinus was found to be higher than the left across all percentages and among all groups -- in both group *a* where the affected sinus is located ipsilateral to the dominant sinus and in group *b* where the affected sinus is located heterolateral to the dominant sinus. Results in all cases indicated right side dominance of the sigmoid sinus.

**Table 2:** Section area of the left and right sigmoid sinus.

	*N* (%)	5%	25%	50%	75%	95%
Total	278 (100)					
Left ethyl sinus (mm^2^)		15.00	31.00	44.00	62.25	93.00
Right ethyl sinus (mm^2^)		24.00	46.00	61.50	82.00	129.05
Ipsilateral group^a^	143 (51.4)					
Left ethyl sinus (mm^2^)		15.20	30.00	42.00	62.00	93.80
Right ethyl sinus (mm^2^)		26.00	45.00	61.00	85.00	139.40
Heterolateral group^b^	135 (48.6)					
Left ethyl sinus (mm^2^)		13.80	34.00	44.00	63.00	91.40
Right ethyl sinus (mm^2^)		21.80	46.00	64.00	80.00	115.00

a Affected sinus is located ipsilateral to the dominant sinus.

b Affected sinus is located heterolateral to the dominant sinus.

The difference between the sides was not statistically significant (*p* > 0.05), however, the section areas of all groups yielded the same result that the right sigmoid sinus takes up higher percentages than the left. Right-side dominance was observed in both group *a* and *b.* This result was brought to attention when determining the location and length of the incision during preoperative preparation to protect the sigmoid sinus (see Table 3).

**Table 3:** Comparison of section area of the left and right sigmoid sinus between the ipsilateral group and heterolateral group.

	Ipsilateral group^a^ Median (IQR)	Heterolateral group^b^ Median (IQR)	*p*
Total			
Left ethyl sinus (mm^2^)	42.00 (30.00, 62.00)	44.00 (34.00, 63.00)	0.576
Right ethyl sinus (mm^2^)	61.00 (45.00, 85.00)	64.00 (46.00, 80.00)	0.627
Left dominant sinus group			
Left ethyl sinus (mm^2^)	64.00 (54.00, 80.00)	66.00 (52.00, 81.00)	0.464
Right ethyl sinus (mm^2^)	45.00 (31.00,50.00)	42.00 (25.50,53.50)	0.784
Right dominant sinus group			
Left ethyl sinus (mm^2^)	34.00 (28.00,47.00)	38.00 (28.00,48.00)	0.714
Right ethyl sinus (mm^2^)	74.50 (53.00, 92.00)	69.50 (54.50, 86.75)	0.348

a Affected sinus is located ipsilateral to the dominant sinus.

b Affected sinus is located heterolateral to the dominant sinus.

### Results of the improved method

We thereby improved the approach of line drawing in the subsequent treatments. The conventional drawing method was to determine the position of the transverse sinus and then draw the line according to the location of the mastoid root to design the surgical incision. In the improved method, having the relationship between the affected side and the dominant side determined, the location of incision was adjusted towards the dorsal if the relationship was ipsilateral, and was adjusted towards the ventral if the relationship was heterolateral, according to the thickness of the skull. Statistical data from 60 treated cases using the improved method are presented in Table 4.

**Table 4:** Comparison of parameters between the ipsilateral group and heterolateral group in 60 cases.

	Ipsilateral group^a^ (Median (IQR) Mean ± SD)	Heterolateral group^b^ (Median (IQR) Mean ± SD)	*t*	*p*
Left ethyl sinus (mm^2^)	54.00 (41.00,83.00)	56.00 (39.00,77.00)	0.259	0.797
Right ethyl sinus (mm^2^)	68.00 (51.00,93.00)	71.00 (55.50,93.50)	0.091	0.928
Angle of Clamp (°)	21.81 ± 4.946	21.4 ± 5.88	0.276	0.784
Depth (mm)	11.87 ± 2.11	12.41 ± 3.72	−0.656	0.515
Anterior bone thickness (mm)	11.37 ± 3.00	11.40 ± 2.49	−0.037	0.971
Posterior bone thickness (mm)	5.18 ± 1.49	5.52 ± 1.72	−0.751	0.456
Pneumatome (mm)	4.84 ± 5.92	0.13 ± 4.58	2.577	0.014
Cerebral compression (mm)	437.19 ± 72.80	429.48 ± 80.45	0.363	0.718
Bone width (mm)	17.70 ± 3.25	16.67 ± 2.97	1.192	0.239
Bone window width(mm)	28.60 ± 3.05	27.35 ± 3.07	1.464	0.150

a Affected sinus is located ipsilateral to the dominant sinus.

b Affected sinus is located heterolateral to the dominant sinus.

According to Table 4, when the affected side was located ipsilateral to the dominant side, the sigmoid sinus was found to be shallower located, and the skull appeared thinner. On the other hand, when the affected side was located heterolateral to the dominant side, the location of sigmoid sinus tended towards the ventral side of the bone window and was found deeper.

**Figure 2:**
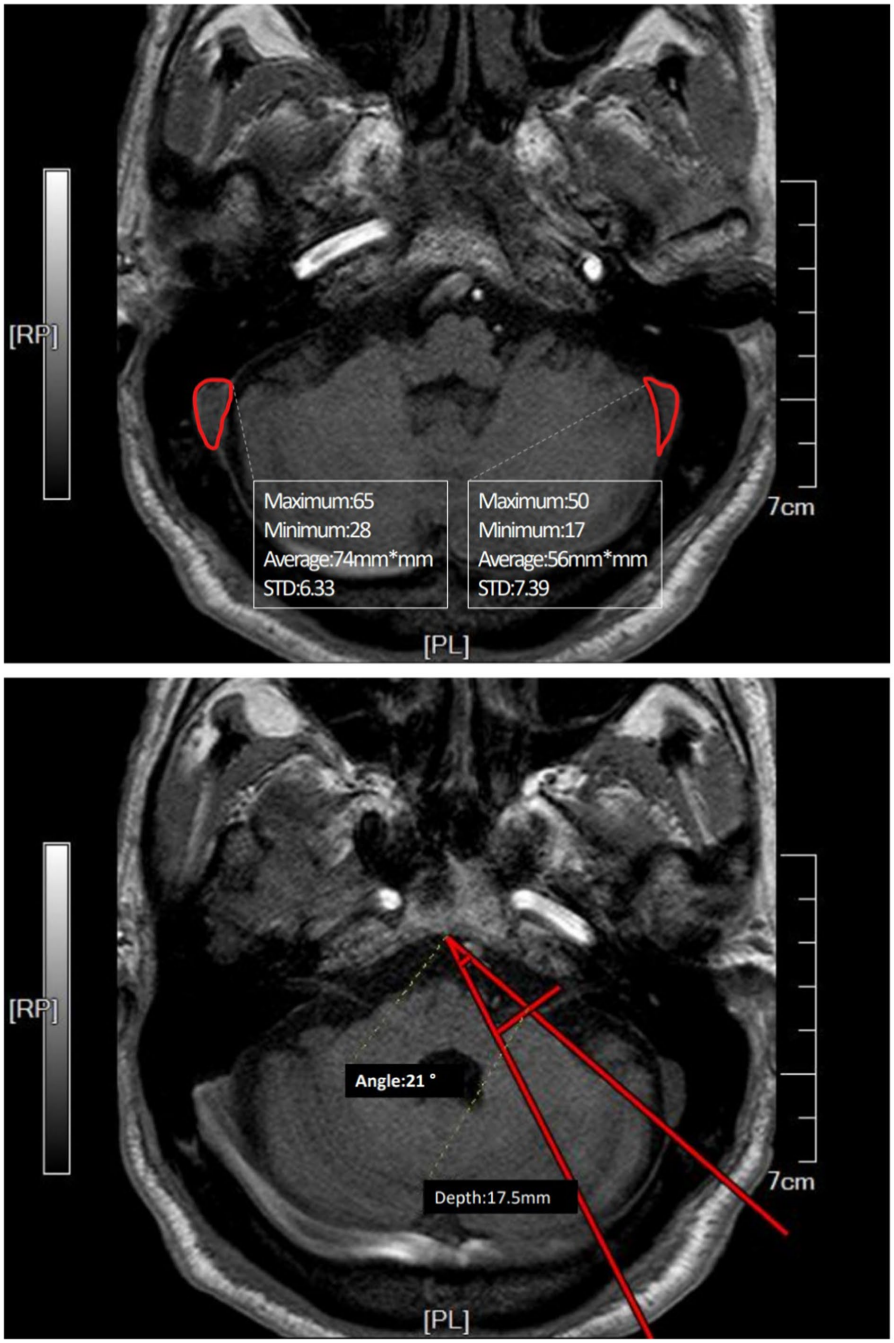
Preoperative MRI image of a case where the affected side and the dominant side are ipsilateral (right side). The area of the sinus was measured in the plane of REZ (root entry zone) where the root of the facial nerve is located. The angle was measured by connecting the midpoint of the REZ plane slope and the sinus border to the responsible blood vessel at its deepest point. The distance from the responsible blood vessel’s deepest point to the perpendicular line from the deepest point of the angle to the petrous bone wall was the depth of the responsible blood vessel.

**Figure 3:**
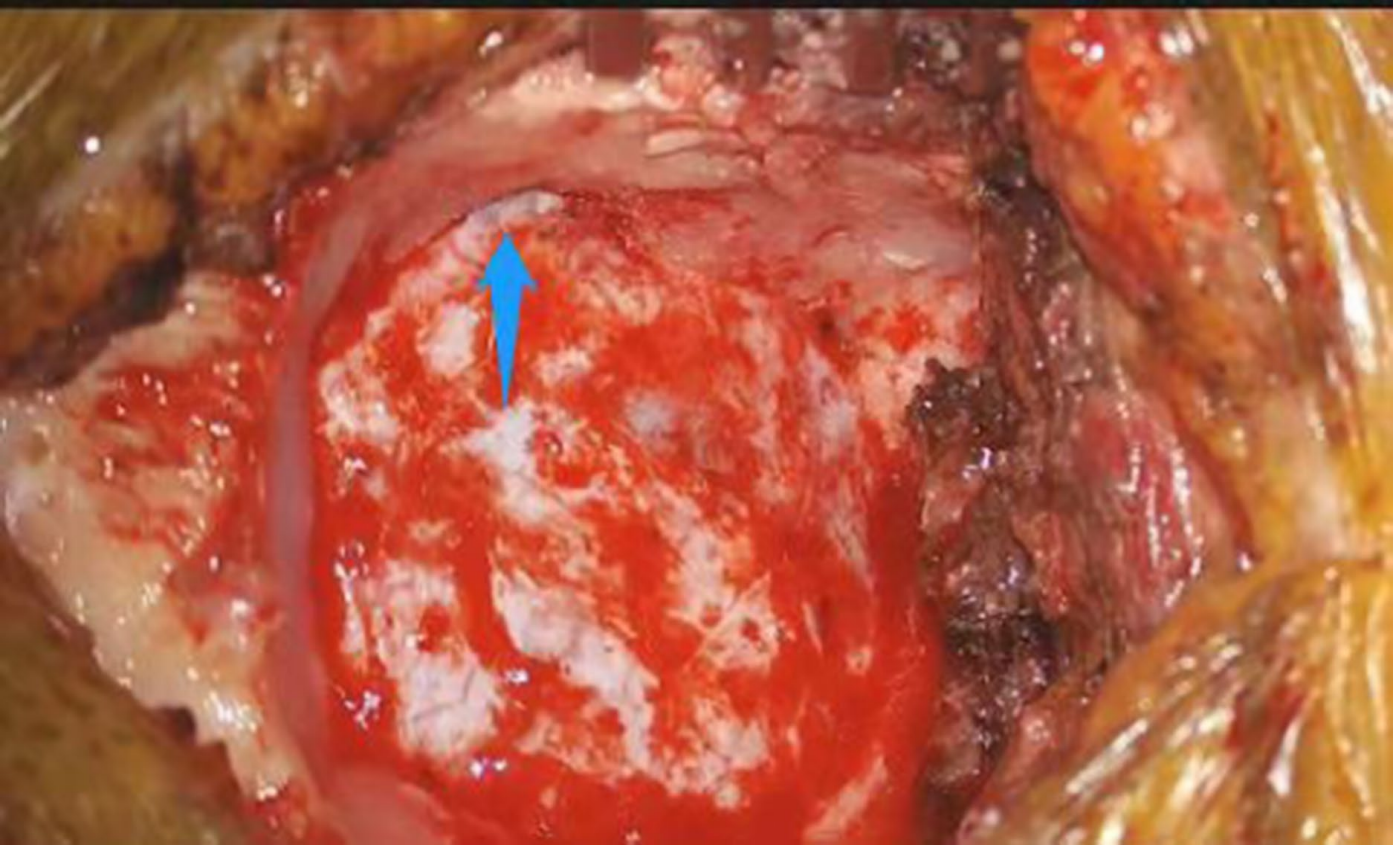
View of sigmoid sinus (pointed by arrow) during retrosigmoid approach on the right hemisphere.

**Figure 4:**
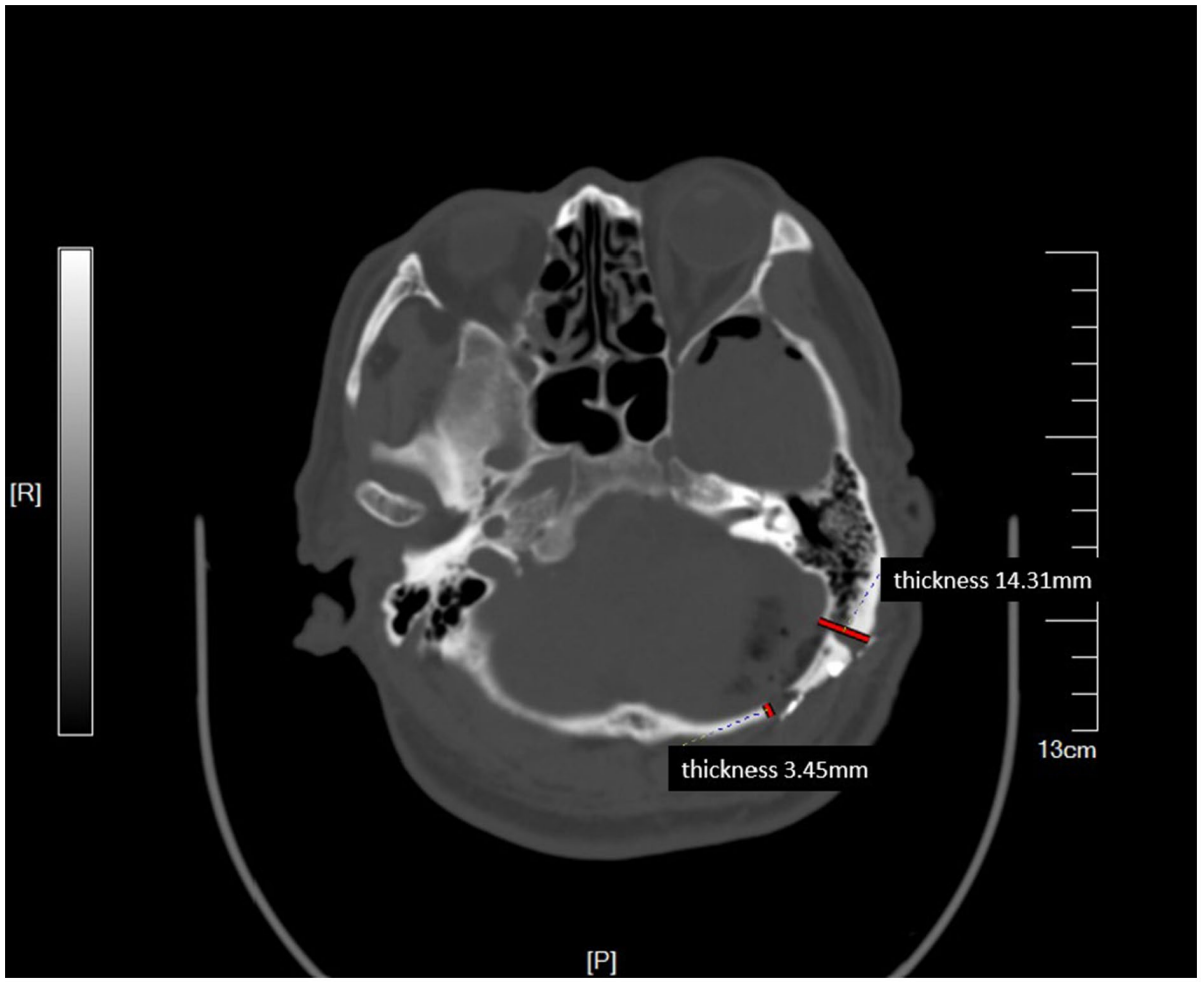
Postoperative CT showing the location of bone flap flush with the sigmoid sinus. The thickness of skull before (45) and behind the bone flap is shown in milimeters.

The difference between the two groups was not statistically significant, given the limited sample size, but the advantage of the improved method based on these differences was evident. Planning the line of incision carefully based on different sizes and angles of sinuses achieved satisfactory surgical view. Intraoperative adjustments of bone flaps due to sinus protection were avoided. Figure 2 shows the preoperative planning MRI image of an improved MVD surgery implementing the improved method. The position and skull thickness of the sigmoid sinus were determined by MRI. The surgical approach for the same case during the surgery is shown in Figure 3, where a bone window is opened to reach the responsible blood vessels, revealing the sigmoid sinus during surgery. The postoperative CT of this case (Figure 4) showed that the bone flap was returned right next to the sinus edge. This way, the risk of damaging venous sinuses was reduced and bone window exposure was improved, offering good surgical fields.

**Figure 5:**
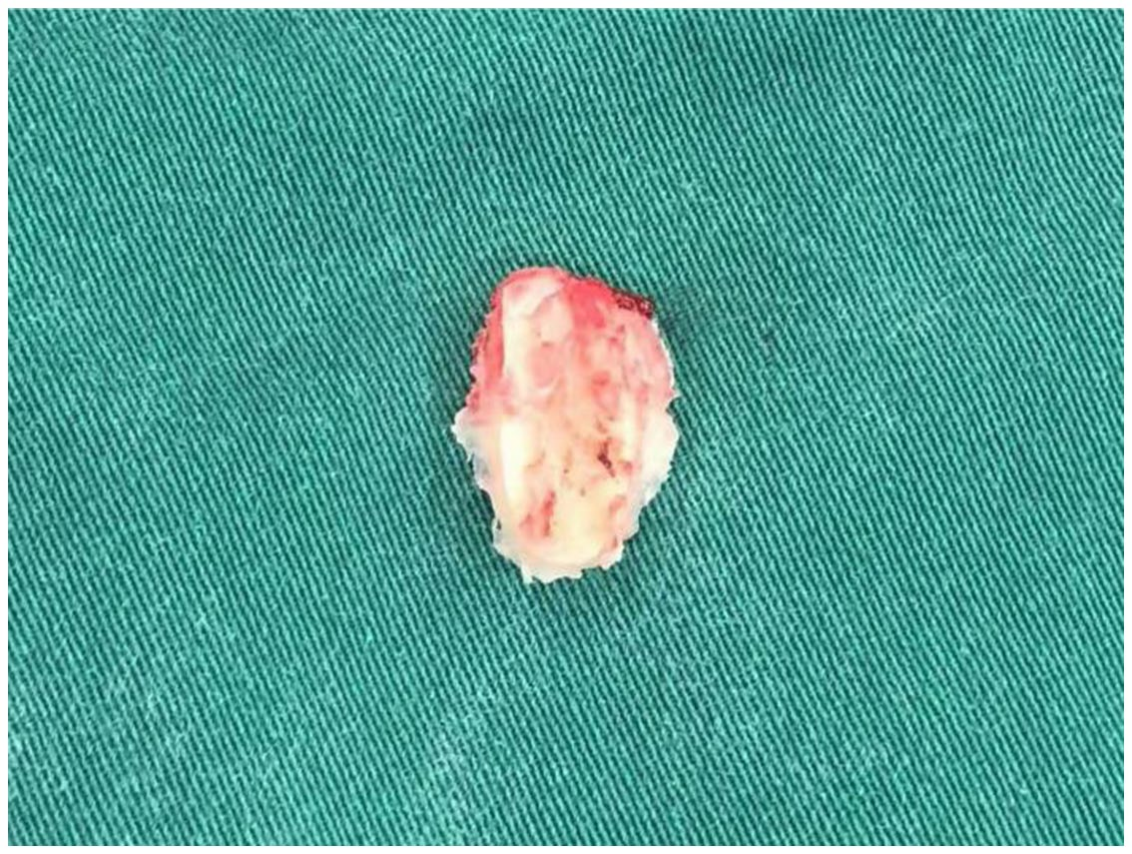
Bone flaps before improvements. Adjustment for a larger bone flap was made due to a deeply located sigmoid sinus, the bone window was narrow and the surgical field was restricted.

**Figure 6:**
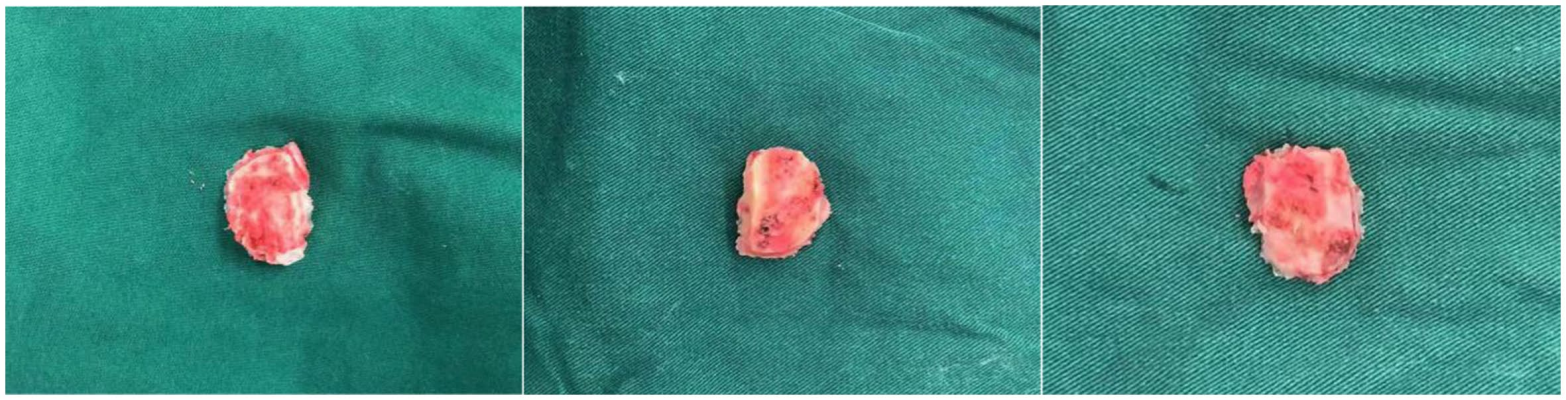
Bone flaps using improved methods. Intraoperative adjustments in bone flap dimensions were avoided.

Comparing the bone flaps before and after implementing the improved method (Figures 5, 6), the advancements were evident. Better surgical views were achieved and intraoperative adjustments of bone flaps were avoided in all cases.

## Discussion

Microvascular decompression relieves abnormal compression of a cranial nerve causing trigeminal neuralgia by isolating the trigeminal nerve root from the offending artery triggering the pain signals (Patel and Liu, 2016). It is a non-destructive surgery that maintains neural function and provides the most prolonged duration of pain relief from trigeminal neuralgia (Sun et al., 2014). Destruction of the venous structure during surgery, especially at the dominant side, may lead to cerebral venous sinus occlusion, cerebral hemorrhage, thrombosis, infarctions and dural arteriovenous malformations (Arnautović et al., 1998; Ohata et al., 1998). Located in the sigmoid groove of the temporal bone, sigmoid sinus receives blood from the transverse sinus from its lateral end. The transverse sinus, as a component of the internal jugular vein system, is mainly responsible for receiving venous blood supply from the inferior cerebral and cerebellar veins (Uzmansel et al., 2013). Given that the exposure of sigmoid sinus during MVD craniotomy marks the completion of the bone flap, structural and positional analysis of it helps the surgeon to predict the dimensions of the bone flap. Using advanced techniques, the surgical field can be improved, and the risk of damaging the sigmoid sinus can be reduced. MVD operation on the side of a wide sigmoid sinus has greater difficulty, for it confines the exposure of bone window, which is often limits the surgical view during interventions. As a result, preoperative design is considered vital in reducing surgical difficulty and the possibility of complications. Analysis of sigmoid sinus sectional area of 278 patients revealed a right-side dominance pattern whenever the affected side is ipsilateral or heterolateral to the dominant sinus. This result coincides with the findings of past year’s dissection study by directly measuring human sigmoid sinuses in cadavers (Aly and Tubbs, 2019; Ozalp et al., 2019) and the findings from a calculation of the sectional areas of 102 samples using MR examination (Manara et al., 2010). From a physiological perspective, blood from the deeper parts of the brain flows through the straight sinus and opens the left transverse sinus which drains into the left sigmoid sinus, which usually smaller; the right transverse sinus receives blood from the superficial parts of the brain by the superior sagittal sinus and drains into the right sigmoid sinus, whose lumen is wider due to the flow volume (Aly and Tubbs, 2019).

**Figure 7:**
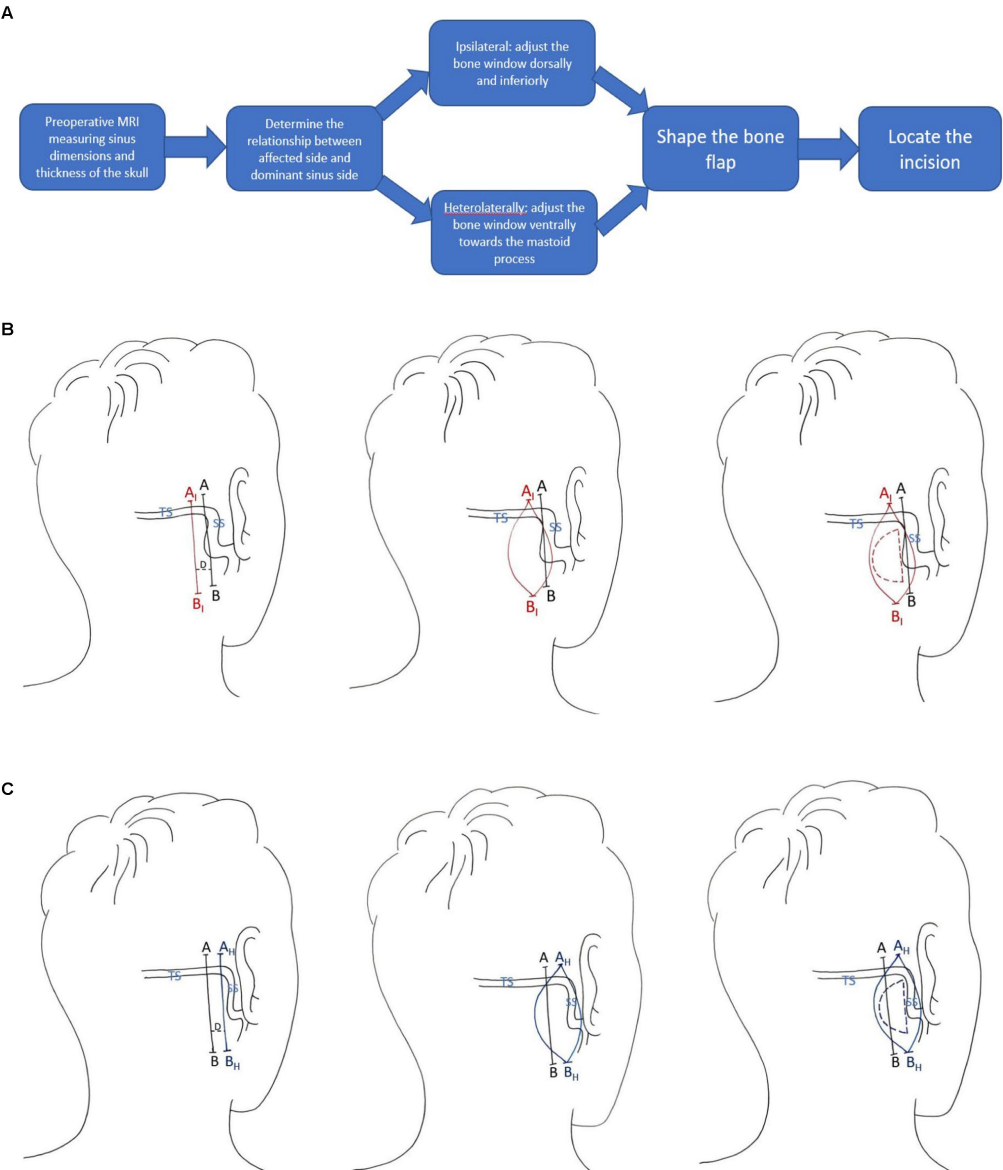
**(A)** Procedure of the improved approach. **(B)** Adaption for ipsilateral cases. Line AB is the conventional drawing and A_I_B_I_ is the improved drawing. The distance D between line AB and A_I_B_I_ should be determined from sigmoid sinus dimensions by case as measured from preoperative MRI. **(C)** Adaption for heterolateral cases. Line AB is the conventional drawing and A_H_B_H_ is the improved drawing. The distance D between line AB and A_H_B_H_ should be determined from sigmoid sinus dimensions by case as measured from preoperative MRI.

The right dominant sigmoid sinus requires careful treatment during surgery. Preoperative design of the bone flap is a practical approach to prevent adjustment of bone flap dimensions. During operations, anatomical characteristics of venous sinuses can vary among individuals and should be identified carefully to avoid destruction to venous sinuses and restriction of bone window exposure (Tomasi et al., 2022). Clinical evidence showed that intraoperative adjustments of bone flaps are accompanied by restrictions to surgical fields that impede operations and can lead to increased tension on nerve bundles of the cerebellar hemisphere, which increases the risk of haemorrhage and contusion of the cerebellum (Kitamura et al., 2017; Wang et al., 2022). Meanwhile, the facial nerve and its branches should be preserved carefully during retrosigmoid craniotomy. Serving as an anatomic landmark of distinguishing the trigeminal nerve, the relative position of sigmoid sinus to the trigeminal nerves is frequently referred to during operation. Improper treatments that result in neurologic damage can give rise to significant complications such as postoperative aphasia and hearing loss (Jackler et al., 1995; Toda et al., 2015). Therefore, preoperative planning by measuring the sigmoid sinus’s location and structure using MRI is recommended. In the improved method, we first measured the position and dimensions of the sigmoid sinus and the thickness of skull at the back and front before shaping the bone flap and designing the incision. Knowing the right dominance pattern of sigmoid sinus, in cases where the affected side was ipsilateral to the dominant sinus, we adapted the incision dorsally and inferiorly to broaden the surgical view and to avoid destroying the sigmoid sinus located in front of the bone window or the transverse sinus located shallowly beneath the skull. In cases where the affected side was heterolateral to the dominant sinus, the sigmoid sinus was observed to lie more ventrally and deeper to the sull, we therefore adapted the incision ventrally to reduce tension on the cerebellum and to achieve better surgical fields without widening the bone window. The results show that the improved approach maximizes the exposure of the bone flap to the sinus edge. Postoperative CT scans show that the edge of the bone window is located at the sinus edge and the bone flap is retracted towards the sinus edge, achieving a better angle for the bone window compared to conventional methods. This effectively avoids the need to adjust the bone flap during surgery to protect the sigmoid sinus (see Figure 7).

It should be noted that dominant pattern of the sigmoid sinus is based on the existing MRI records at our clinic, no statistical significance indicates limitation in the sample size. The pattern should be further validated in a larger cohort.

In conclusion, surgical planning before MVD operation is essential to protect venous structures. Based on the right dominance pattern of sigmoid sinus, MRI examination serves to determine the relationship between the affected side and the dominant side in evaluating the positional and structural characteristics of the sigmoid sinus by individuals, to facilitate the retention of facial acoustic nerve in MVD surgery and to reduce the risk of glossopharyngeal nerve injury and cerebellum contusion. Such designs and improvements have yielded lower risks and good prognoses.

## Data availability statement

The original contributions presented in the study are included in the article/supplementary material, further inquiries can be directed to the corresponding authors.

## Author contributions

CZ and YC contributed equally to the design and drafting of the manuscript. CjJ was in charge of data analysis and literature review. WX, JW, CgJ, and BW revised the manuscript. WL monitored the quality and integrity of this work. All authors contributed to the article and approved the submitted version.

## Conflict of interest

The authors declare that the research was conducted in the absence of any commercial or financial relationships that could be construed as a potential conflict of interest.

## Publisher’s note

All claims expressed in this article are solely those of the authors and do not necessarily represent those of their affiliated organizations, or those of the publisher, the editors and the reviewers. Any product that may be evaluated in this article, or claim that may be made by its manufacturer, is not guaranteed or endorsed by the publisher.

## References

[ref1] Aly I. Tubbs R. S. (2019). “Chapter 5 the sigmoid sinus” in Anatomy, Imaging and Surgery of the Intracranial Dural Venous Sinuses. 1st ed (Elsevier), 47–58.

[ref2] Apra C. Lefaucheur J.-P. Le Guérinel C. (2017). Microvascular decompression is an effective therapy for trigeminal neuralgia due to dolichoectatic basilar artery compression: case reports and literature review. Neurosurg. Rev. 40, 577–582. doi: 10.1007/s10143-017-0812-5 28091827

[ref3] Arnautović K. I. Al-Mefty O. Angtuaco E. Phares L. J. (1998). Dural arteriovenous malformations of the transverse/sigmoid sinus acquired from dominant sinus occlusion by a tumor: report of two cases. Neurosurgery 42, 383–388. doi: 10.1097/00006123-199802000-00112 9482191

[ref4] Ozalp H. Aktekin M. Hamzaoglu V. Vayisoglu Y. Karatas M. A. Karsiyaka D.. (2019). The comparison of the right and left sigmoid sinus cross-sectional areas in fetal period and the factors affecting the venous dominance. J.Int. Adv. Otol. 15, 409–414. doi: 10.5152/iao.2019.5876 31846921 PMC6937183

[ref5] Jackler R. K. Sim D. W. Gutin P. H. Pitts L. H. (1995). Systematic approach to intradural tumors ventral to the brain stem. Am. J. Otol. 16, 39–51. 8579176

[ref6] Kitamura M. A. P. Costa L. F. Silva D. O. d. A. Batista L. L. Holanda M. M. d. A. Valença M. M. (2017). Cranial venous sinus dominance: what to expect? Analysis of 100 cerebral angiographies. Arq. Neuropsiquiatr. 75, 295–300. doi: 10.1590/0004-282x20170042 28591389

[ref7] Lee M. H. Lee J.-A. Park K. (2019). Different roles of microvascular decompression in Hemifacial spasm and trigeminal neuralgia. J. Neurol. Surg. B Skull Base 80, 511–517. doi: 10.1055/s-0038-1676377 31534894 PMC6748867

[ref8] Manara R. Mardari R. Ermani M. Severino M. Santelli L. Carollo C. (2010). Transverse dural sinuses: incidence of anatomical variants and flow artefacts with 2D time-of-flight MR venography at 1 tesla. Medicine 115, 326–338. doi: 10.1007/s11547-010-0480-9 20058094

[ref9] Min L. Liu M. Zhang W. Tao B. Sun Q. Li S.. (2019). Outcomes and safety of overlapping surgery in patients undergoing microvascular decompression for Hemifacial spasm and trigeminal neuralgia. World Neurosurg. 130, e1084–e1090. doi: 10.1016/j.wneu.2019.07.092 31323398

[ref10] Møller A. (1998). Vascular compression of cranial nerves. I. History of the microvascular decompression operation. Neurol. Res. 20, 727–731. doi: 10.1080/01616412.1998.11740591 9864738

[ref11] Montava M. Rossi V. Curto Fais C. L. Mancini J. Lavieille J.-P. (2016). Long-term surgical results in microvascular decompression for hemifacial spasm: efficacy, morbidity and quality of life. Acta Otorhinolaryngol. Ital. 36, 220–227. doi: 10.14639/0392-100X-899 27214834 PMC4977010

[ref12] Ohata K. Haque M. Morino M. Nagai K. Nishio A. Nishijima Y.. (1998). Occlusion of the sigmoid sinus after surgery via the presigmoidal-transpetrosal approach. J. Neurosurg. 89, 575–584. doi: 10.3171/jns.1998.89.4.0575 9761051

[ref13] Patel S. K. Liu J. K. (2016). Overview and history of trigeminal neuralgia. Neurosurg. Clin. N. Am. 27, 265–276. doi: 10.1016/j.nec.2016.02.002 27324994

[ref15] Sun J. Li J. Deng X. Long J. (2014). Clinical treatment progress of trigeminal neuralgia. Prog. Modern Biomed. 14, 5189–5193.

[ref16] Toda H. Goto M. Iwasaki K. (2015). Patterns and variations in microvascular decompression for trigeminal neuralgia. Neurol. Med. Chir. 55, 432–441. doi: 10.2176/nmc.ra.2014-0393 PMC4628171 25925756

[ref17] Tomasi S. O. Umana G. E. Scalia G. Raudino G. Graziano F. Palmisciano P.. (2022). The superficial anastomosing veins of the human brain cortex: a microneurosurgical anatomical study. Front. Surg. 8:817002. doi: 10.3389/fsurg.2021.817002 35083275 PMC8784509

[ref18] Tomasi S. O. Umana G. E. Scalia G. Rubio-Rodriguez R. L. Cappai P. F. Capone C.. (2020). Importance of veins for neurosurgery as landmarks against brain shifting phenomenon: an anatomical and 3D-MPRAGE MR reconstruction of superficial cortical veins. Front. Neuroanat. 14:596167. doi: 10.3389/fnana.2020.596167 33384587 PMC7771049

[ref19] Uzmansel D. Kurtoğlu Z. Talas D. Dağtekin A. Avcı E. Karataş M. (2013). Evaluation of cross-sectional areas of the sigmoid sinus, jugular bulb and internal jugular vein: a cadaver study. J. Int. Adv. Otol. 9:240.

[ref20] Van Osch K. Allen D. Gare B. Hudson T. J. Ladak H. Agrawal S. K. (2019). Morphological analysis of sigmoid sinus anatomy: clinical applications to neurotological surgery. J. Otolaryngol. Head Neck Surg. 48:2. doi: 10.1186/s40463-019-0324-0. 30635049 PMC6329078

[ref21] Wang J. Chong Y. Jiang C. Dai Y. Liang W. Ding L. (2022). Microvascular decompression for hemifacial spasm involving the vertebral artery. Acta Neurochir. 164, 827–832. doi: 10.1007/s00701-021-05076-8 34870744 PMC8913562

